# The Short-Term Impact of Decompressive Craniectomy in Pediatric Patients with Severe Traumatic Brain Injury: A Retrospective Matched Cohort Study

**DOI:** 10.3390/children12101374

**Published:** 2025-10-11

**Authors:** Jingjing Xu, Run Zhou, Jing Li, Chengjun Liu, Hongxing Dang

**Affiliations:** 1 Department of Pediatric Intensive Care Unit, Ministry of Education Key Laboratory of Child Development and Disorders, Children’s Hospital of Chongqing Medical University, Chongqing 400014, China; 2 National Clinical Research Center for Child Health and Disorders, China International Science and Technology Cooperation Base of Child Development and Critical Disorders, Chongqing 400014, China; 3 China International Science and Technology Cooperation Base of Child Development and Critical Disorders, Chongqing 400014, China

**Keywords:** traumatic brain injury, decompressive craniectomy, children, prognosis, intensive care

## Abstract

**Highlights:**

**What are the main findings?**

- In a 1:1 matched cohort (53 DC; 53 non-DC), decompressive craniectomy did not reduce in-hospital mortality (17.0% vs. 26.4%, *p* = 0.239) or resource use (duration of ventilation, ICU stay, total hospital stay; all *p* > 0.05).
- DC was associated with better neurological status at discharge (PCPC, *p* = 0.029); the difference at 3 months was not statistically significant but showed a near-significant trend (*p* = 0.057).

**What are the implications of the main findings?**

- For children with severe TBI who meet surgical criteria, DC may confer short-term neurological benefits without improving short-term survival or length of stay—useful for family counseling and perioperative expectation-setting.
- Confirmation requires adequately powered prospective studies with standardized ICP/CPP monitoring and longer follow-up to determine durability and to identify subgroups most likely to benefit.

**Abstract:**

**Background/Objectives**: Decompressive craniectomy (DC) is commonly applied to manage refractory intracranial hypertension in severe traumatic brain injury (TBI). However, its role and benefits in pediatric populations remain uncertain. Clarifying whether DC provides measurable clinical advantages in children with severe TBI may inform treatment strategies and family counseling. **Methods**: We conducted a retrospective, one-to-one matched cohort study at a tertiary pediatric center (2014–2023). Fifty-three children with severe TBI who underwent DC were matched with fifty-three non-DC patients based on age, Glasgow Coma Scale score, cranial CT findings, and pupillary response at admission to ensure comparable injury severity. Demographic data, clinical features, and outcomes were collected. Primary outcomes were in-hospital mortality and Pediatric Cerebral Performance Category (PCPC) scores at discharge and 3 months. Secondary outcomes included duration of mechanical ventilation, intensive care unit (ICU) stay, and total hospital stay. **Results**: Mortality did not differ significantly between DC and non-DC groups (17.0% vs. 26.4%, *p* = 0.239). DC patients had better PCPC scores at discharge (*p* = 0.029). At 3 months, the between-group difference was not statistically significant but showed a near-significant trend (*p* = 0.057). No significant differences were observed in duration of ventilation (*p* = 0.100), ICU stay (*p* = 0.348), or hospital stay (*p* = 0.678). **Conclusions**: DC may not reduce short-term mortality in pediatric severe TBI but appears to be associated with more favorable neurological outcomes at discharge. Larger, adequately powered studies with standardized monitoring and longer follow-up are needed to clarify the durability and scope of potential benefits in this population.

## 1. Introduction

Traumatic brain injury (TBI) is a leading cause of permanent disability and mortality among children, exerting a profound impact on the children, their families, and society [1]. The global incidence of TBI in the pediatric population ranges from approximately 47 to 280 per 100,000 children [1]. Pathological changes, including cerebral contusion, edema, and intracranial hemorrhage following TBI, can result in elevated intracranial pressure (ICP). This increase in ICP can exacerbate neurological injury, lead to cerebral herniation, and potentially result in brain death once a critical threshold is surpassed [2]. Prior research has demonstrated that persistent intracranial hypertension exceeding 20 mmHg in children with TBI is associated with a poor prognosis and elevated mortality rates [3,4]. Decompressive craniectomy (DC), as a therapeutic strategy for refractory intracranial hypertension, serves to increase intracranial volume, thereby reducing ICP, enhancing cerebral perfusion pressure (CPP), improving oxygenation, and augmenting the compliance of brain tissue, which can decrease the incidence of brainstem compression [5,6,7,8].

There remains a debate regarding the utilization of DC in the treatment of children with TBI. On one hand, children possess more elastic brain tissue and a relatively greater volume of cerebrospinal fluid, providing a larger intracranial compensatory capacity compared to adults, which may increase the likelihood of successful conservative management. On the other hand, children often experience severe cerebral edema following TBI and are susceptible to rapid brain herniation; in such cases, early DC might offer potentially reduce mortality [9].

While the judicious application of DC can be life-saving, it also carries the risk of inflicting significant secondary damage to the central nervous system [10]. Current clinical guidelines are hesitant to advocate for DC as a primary treatment modality for TBI in children, primarily due to the scarcity of clinical studies specifically addressing DC in the pediatric context [11].

This study aimed to analyze the clinical characteristics and postoperative outcomes in children with severe TBI. Specifically, it compared in-hospital mortality and short-term neurological outcomes at discharge between children who underwent DC and those managed without DC. The goal was to inform clinical decision-making regarding DC in pediatric TBI and assist in prognostic communication with families.

## 2. Methods

This study collected data on a cohort of children who were hospitalized for severe TBI at the Children’s Hospital of Chongqing Medical University between March 2014 and March 2023 and who met the predefined clinical indications for DC. Inclusion was consecutive. Patients who ultimately underwent DC during hospitalization were assigned to the DC group. To form a 1:1 matched control group, children from the same cohort who met the surgical indications but did not receive DC were selected using a combination of algorithm-based and manual matching based on established clinical criteria.

**Inclusion criteria:** Age 18 years or younger; admission within 24 h post-TBI; a Glasgow Coma Score (GCS) of 8 or lower upon admission; had indications for DC surgery; children in the DC group had undergone DC during their hospital stay, whereas those in the non-DC group may have received other brain surgeries excluding DC.

**Exclusion criteria:** Pre-existing neurological disorders prior to TBI, and death within 24 h of hospital admission.

**Matching criteria:** Matching criteria were applied to identify control patients from a retrospective cohort of 186 children who met the surgical indications for DC but did not undergo the procedure. Initial screening was performed using structured database queries to pre-select candidates based on predefined parameters. Final 1:1 matches were manually confirmed to ensure optimal comparability.

Matching variables included the following: (1) age difference within 2 years; (2) GCS score on admission ≤ 8; (3) pupillary light reflex on admission being similar, either bilaterally absent or severely diminished; (4) CT comparability was assessed using a predefined numeric concordance scoring system. Nine major CT findings were evaluated: subarachnoid hemorrhage (SAH), subdural hematoma (SDH), epidural hematoma (EDH), cerebral herniation, midline shift, intraventricular hemorrhage, cerebellar hematoma, diffuse axonal injury (DAI), and cerebral edema. Cerebral herniation, midline shift, and DAI were assigned 2 points each, while the others were assigned 1 point each, giving a maximum score of 12. Two patients were considered adequately matched when at least five out of nine findings were concordant, corresponding to a score ≥ 8/12. CT scans were independently reviewed by two investigators, with disagreements resolved by a senior pediatric neurosurgeon.

**Data collection:** Demographic information and medical history were collected, encompassing age, gender, the mechanism of injury, the time interval from injury to hospital admission, GCS, cerebral CT scan results, mean arterial blood pressure upon admission, and the presence of spinal cord injury, posttraumatic seizures, coagulopathy, or multiple traumas. Multiple traumas were defined as the presence of traumatic injuries involving at least two distinct organ systems, confirmed by clinical and radiological evaluation. This included thoracic and abdominal organ injuries, long bone fractures, and pelvic fractures. Treatment data included the side and site of the bone flap in the DC group, postoperative complications, follow-up surgeries, and medical interventions such as hyperosmolar therapy, sedation, analgesia, phenobarbital administration, and blood transfusion for both groups. Clinical outcomes assessed included mortality, PCPC [12] at discharge and three months post-discharge, duration of mechanical ventilation, length of ICU stay and total hospital stay.

**DC and postoperative care:** All children included in this study (both DC and non-DC groups) had indications for DC before surgery. At least two attending neurosurgeons jointly recommended DC to the guardians. This recommendation primarily followed the guidelines of the expert consensus on childhood craniocerebral injury, which suggest considering DC for children with TBI who exhibit significant neurological deterioration, the presence of cerebral herniation, and unresponsiveness to standard medical interventions aimed at reducing intracranial pressure [2].

In our institution, DC was performed as a standardized procedure involving removal of a large frontotemporoparietal bone flap, typically measuring at least 12 cm × 10 cm. The dura was opened in a cruciate or stellate fashion, and artificial duraplasty was routinely performed.

The final decision on whether to proceed with DC was made by the child’s guardians after they were fully informed. Considering the uncertainties regarding the risks and efficacy of DC, the guardians had the right to either consent to or refuse DC, and they signed a written informed consent form.

All procedures performed by the same neurosurgeon team at our hospital. Postoperatively, all children were transferred to the Pediatric Intensive Care Unit (PICU) for close monitoring and treatment. In cases where ICP monitoring was implemented, ICP data were recorded every 2 h and when the value increased abnormally by the PICU nursing staff. CPP was calculated using the ICP and mean arterial pressure at the time of recording.

**Results Record** Mortality and Pediatric Cerebral PCPC at discharge were used as the primary outcomes [12]. A favorable outcome was defined as good or mild disability (PCPC1-2), while a poor outcome was defined as severe disability, vegetative state or coma, and death (PCPC3-6). The PCPC of each child was assessed by PICU attending physicians. Secondary outcomes were duration of mechanical ventilation, length of stay in ICU, and length of stay in hospital, and PCPC 3 months after discharge.

**Statistical Analysis** Statistical analysis followed the SAMPL guidelines and was performed using SPSS version 25.0. Rank variables were expressed as medians with interquartile ranges (IQR) and compared using the Wilcoxon signed-rank test. Continuous variables were presented as mean ± standard deviation or median, depending on their distribution, and compared using the paired Student’s *t*-test or the Wilcoxon signed-rank test. Categorical variables were summarized as counts (percentages) and compared primarily using McNemar’s test within matched pairs; chi-square tests were used as appropriate for non-paired exploratory comparisons. All comparisons were conducted within matched pairs to account for the 1:1 matching design. A two-tailed *p*-value < 0.05 was considered statistically significant. The dataset supporting this study is publicly available in Mendeley Data [13].

## 3. Results

### 3.1. Demographic and Medical Characteristics

**Figure 1:**
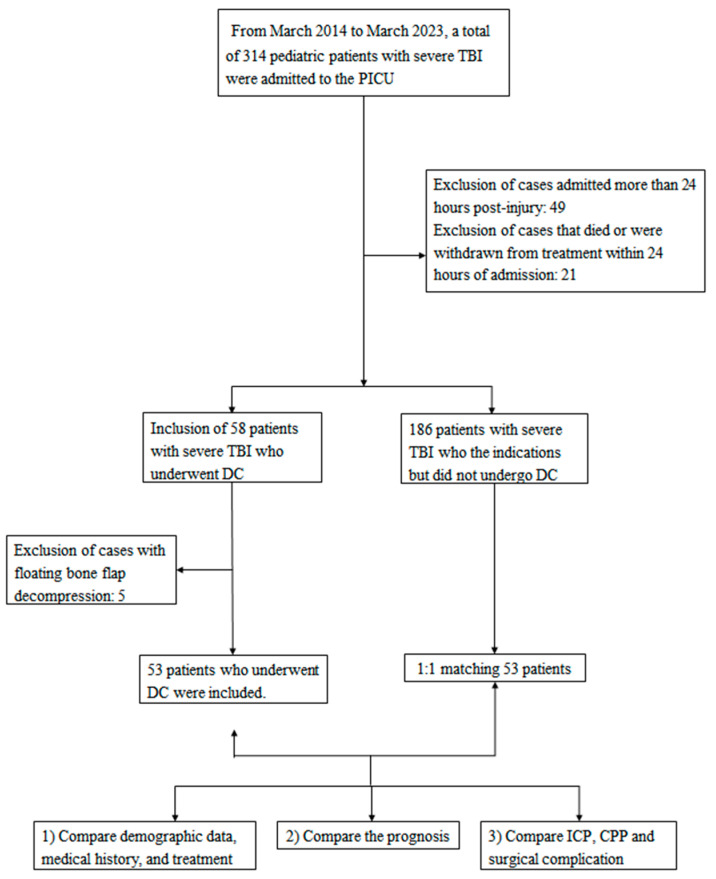
Flowchart of participant inclusion, exclusion, and group matching criteria (STROBE flow diagram). TBI = traumatic brain injury; PICU = Pediatric Intensive Care Unit; DC = Decompressive Craniectomy; ICP = intracranial pressure; CPP = cerebral perfusion pressure.

Over the course of the study period, a total of 314 pediatric patients with severe TBI were admitted to the PICU following surgery. Among these, 53 patients underwent DC. An equal number, 53 out of the 186 children with severe TBI who met the indications but refused and did not undergo DC, were selected and matched on a 1:1 basis as controls (Figure 1). No significant differences were observed between the two groups with respect to demographic information, general condition upon admission, and the medical emergency treatment provided (Table 1). All included patients had complete clinical, imaging, and outcome data, and no cases were excluded due to missing data.

### 3.2. Mortality and Neurological Outcomes

Nine children in the DC group and fourteen children in the non-DC group died in hospital. There was no statistically significant difference in mortality between the two groups (17.0% vs. 26.4%, *p* = 0.239). However, a significantly higher proportion of patients in the DC group demonstrated favorable PCPC scores at discharge compared to the non-DC group (*p* = 0.029) (Table 2). No statistically significant differences were identified between the two groups in terms of the duration of mechanical ventilation, the length of stay in the ICU and in the hospital, or the PCPC scores at the 3-month follow-up assessment.

### 3.3. ICP and CPP

**Figure 2:**
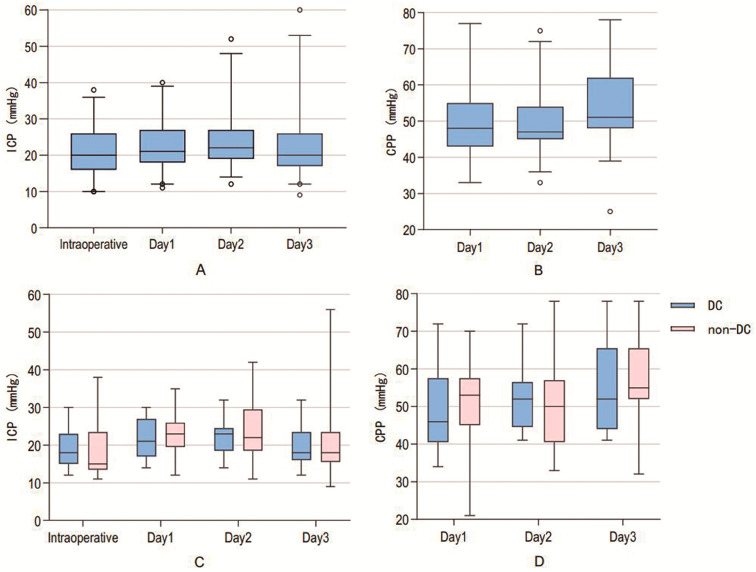
Trends in intracranial pressure (ICP) (**A**) and cerebral perfusion pressure (CPP) (**B**) among all 39 children in the DC group. Comparison of ICP (**C**) and CPP (**D**) between 13 matched pairs of children in the DC and non-DC groups.

A total of 39 children in the DC group and 13 children in the non-DC group had ICP monitored for at least 3 days postoperatively. Firstly, we compared whether there were differences in ICP and CPP across different time periods within the DC group. It was found that postoperative ICP in the DC group exhibited an initial increase followed by a decrease (Figure 2A), while CPP showed the opposite trend (Figure 2B). Notably, on the second and third postoperative days, ICP (22.0 vs. 17.0 mmHg, *p* = 0.042) and CPP (47.0 vs. 51.0 mmHg, *p* < 0.001) within the DC group demonstrated statistically significant differences.

Secondly, we analyzed 13 children in the non-DC group who underwent ICP monitoring. We compared them with 13 corresponding children in the DC group (all of them also underwent ICP monitoring). It was found that the non-DC group exhibited a similar trend with DC group, and a significant difference in CPP was observed on the second and third postoperative days (50.0 vs. 55.0 mmHg, *p* = 0.010). However, further analysis revealed that there were no significant differences in ICP and CPP between the DC and non-DC groups across the various time periods analyzed (Figure 2C,D).

### 3.4. Surgery and Complications

Surgical outcomes for children in the DC group are detailed in Table 3. No significant differences were observed in in-hospital mortality or the rate of poor outcomes at discharge among children based on the side and site of the bone flap removed.

A total of 13 children in the non-DC group underwent various neurosurgical procedures, predominantly including the placement of ICP monitoring probes, hematoma evacuation, dural repair, and lumbar drainage. Postoperative complications, common to both groups, encompassed hemorrhage, hydrocephalus, intracranial infection, and cerebral herniation, as outlined in Table 4.

### 3.5. Follow-Up Surgery

Of the 44 children in the DC group who survived, 31 (70.4%) underwent subsequent surgical procedures at our facility, with an average interval of 2.8 months following DC. These procedures included cranioplasty in 28 cases, Ommaya reservoir implantation in 2 cases, and lumbar drainage in 1 case.

## 4. Discussion

In this cohort study, we used a strict 1:1 matching method to analyze 53 children who underwent DC. Our study indicates that DC was not associated with reduced mortality or sustained improvements in ICP or CPP in children with severe TBI, but may be linked to more favorable short-term neurological outcomes at discharge.

Currently, there is a scarcity of clinical evidence supporting the use of DC as a foundational strategy in the management of pediatric TBI, leading to less definitive surgical recommendations for children [11,14]. A randomized controlled trial, DECRA, examining early DC in patients with TBI demonstrated that in adults with refractory intracranial hypertension, DC was associated with worsened neurological outcomes [15]. Additionally, the multicenter, randomized RESCUEicp trial, which included patients ranging from 10 to 65 years of age, indicated that DC, when compared to non-DC treatments, could effectively reduce mortality rates and improve neurological prognosis. However, this study included only 16 (3.9%) patients under the age of 16, limiting its applicability and guidance for pediatric patients [16]. The average ages of children in the DC and non-DC groups within this study were 5.0 and 4.7 years old, respectively, which is younger than the 5–13 year age range observed in similar studies but older than the average age of 3.2 years for Chinese children with TBI [1].

A study by Rallis, D et al. reported that while salvage DC can effectively control ICP and CPP in children with TBI, long-term neurological outcomes remain unfavorable [17]. The present study observed that children undergoing DC exhibited better neurological status at discharge, although in-hospital mortality did not differ significantly between groups. The mortality rate within the DC group in this study was 17.0%, comparable to the 14.3% observed in the study by Perez et al. [18], and lower than the 20–30% range reported in other studies [17,19,20,21,22,23]. The rate of poor outcomes, defined by PCPC of 3–6, was 52.8% in our study, slightly exceeding that reported in similar studies. This discrepancy may be attributed to varying evaluation standards and the predominance of traffic accidents and falls from height as main mechanisms of injury in our study population. Additionally, no significant differences in brain function were observed between the DC and non-DC groups at the 3-month follow-up, a result that may be attributable to the combined effect of many factors during rehabilitation.

In this study, although the difference in PCPC scores between groups was not statistically significant at 3 months (*p* = 0.057), this value was very close to the conventional α = 0.05 threshold and may still suggest a clinically relevant trend. The early improvement observed at discharge likely reflects the acute benefit of DC in relieving intracranial hypertension. However, post-discharge multidisciplinary rehabilitation in both groups, the inherent neuroplasticity of children, and the limited statistical power of the follow-up analysis may have contributed to convergence of outcomes over time. These observations indicate that early benefits of DC may not automatically translate into durable improvements without sustained rehabilitation support.

A study by Mhanna, M. et al. observed no significant differences in ICP and CPP during the first five postoperative days when comparing 17 pairs of children, one from the DC group and one from the control group [19]. In the current study, continuous ICP monitoring for 3 days post-surgery was conducted in 39 children of the DC group and 13 children of the non-DC group. Postoperative ICP levels were maintained at approximately 20 mmHg, and CPP at approximately 50 mmHg, in both groups. No significant differences in ICP and CPP were identified between the two groups across the monitored time periods. Given the limited number of patients with complete ICP/CPP data in both groups, the ability to detect differences was inherently constrained.

DC is generally applied with the intention of alleviating acute intracranial hypertension, although sustained postoperative normalization of ICP may not be consistently observed in clinical practice. Given that both the primary brain injury and subsequent craniocerebral surgery can result in bleeding and edema, achieving and maintaining absolutely normal ICP levels immediately post-surgery may be challenging.

Common postoperative complications associated with DC encompass hydrocephalus, infection, epileptic seizures, and skin flap depression syndrome [24]. The literature indicates that children who undergo DC have a higher incidence of hydrocephalus compared to those who do not receive DC (15–40%) [24,25,26,27]. But in this study, the main complications of DC were new hematoma, cerebral edema, hydrocephalus, and herniation. These complications did not significantly differ from those observed after other cranial surgeries in the non-DC group.

Prior studies have noted that skull defects in growing children present unique management challenges, potentially complicating the timing and outcomes of skull reconstruction [27]. Prolonged skull defects, however, can impose a psychological burden, potentially hindering the recovery of neurological function [28]. Within this study, 63.6% of the children underwent cranioplasty in our hospital, with an average time to surgery of 3 months post-DC. It is also imperative to conduct psychological evaluations during the follow-up period [23].

Beyond our cohort, recent reviews and randomized trials further underscore the ongoing controversy regarding DC. While it remains an important last-tier therapy for refractory intracranial hypertension, its benefits are highly context-dependent and constrained by notable complication risks [28,29]. Timing also appears critical, with early intervention (<12 h) associated with more favorable outcomes than delayed surgery [30]. Moreover, the most recent large-scale randomized trial found no significant differences in long-term functional outcomes between craniotomy and craniectomy for acute subdural hematoma, though complication profiles differed [31]. Together, these findings highlight the complexity of surgical decision-making and the importance of careful patient selection and postoperative management in children.

This study has several limitations. Firstly, although it is among the few studies focused on pediatric DC, its single-center retrospective design and modest sample size limit the generalizability of the findings. Secondly, despite using rigorous matching criteria, residual confounding cannot be entirely ruled out. Thirdly, the short-term follow-up period may not fully capture long-term neurological outcomes. Therefore, our results should be interpreted as preliminary and hypothesis-generating, rather than definitive. Larger, prospective, multi-center studies are needed to further evaluate the potential role of DC in pediatric TBI and to inform evidence-based guidelines.

## Figures and Tables

**Table 1:** Demographic characteristics, medical histories, and medical treatment modalities for the two groups.

Variable	All Patients (N = 106)	DC Group (n_1_ = 53)	Non-DC Group (n_2_ = 53)	X^2^/z	*p*-Value
Age (yrs)	4.8 (2.8, 7.6)	5.0 (2.8, 8.2)	4.7 (2.8, 7.6)	−1.726	0.084
Sex					
Female	39 (36.8)	20 (37.7)	19 (35.8)	0.041	0.840
Male	67 (63.2)	33 (62.3)	34 (64.2)		
Mechanism of injury					
Traffic accident	45 (42.5)	18 (34.0)	27 (50.9)		0.136
Fall from height	53 (50.0)	29 (54.7)	24 (45.3)	4.378	
Others	8 (7.5)	6 (11.3)	2 (3.8)		
Injury–hospital interval (h)	5.0 (3.4, 9.0)	5.0 (3.3, 7.8)	6.0 (3.5, 11.5)	−1.460	0.144
GCS on admission	6.0 (4.8, 7.0)	7.0 (4.5, 8.0)	6.0 (4.5, 7.0)	−1.781	0.075
CT findings					
SAH	97 (91.5)	49 (92.5)	48 (90.6)	0.000	1.000
SDH	35 (33.0)	23 (43.4)	12 (22.6)	5.161	0.023
EDH	33 (31.1)	19 (35.8)	14 (26.4)	1.100	0.294
Brain herniation	30 (28.3)	18 (34.0)	12 (22.6)	1.674	0.196
Midline shift	4 (3.8)	2 (3.8)	2 (3.8)	0.000	1.000
Intraventricular hemorrhage	37 (34.9)	18 (34.0)	19 (35.8)	0.042	0.839
Cerebellar hematoma	9 (8.5)	4 (7.5)	5 (9.4)	0.000	1.000
DAI	11 (10.4)	5 (9.4)	6 (11.3)	0.101	0.750
Cerebral edema	16 (15.1)	10 (18.9)	6 (11.3)	1.178	0.278
Skull fracture	75 (70.8)	35 (66.0)	40 (75.5)	1.140	0.286
Spinal Injury	13 (12.3)	6 (11.3)	7 (13.2)	0.088	0.767
Traumatic Seizures	37 (34.9)	19 (35.8)	18 (34.0)	0.042	0.839
Coagulopathy	59 (55.7)	31 (58.5)	28 (52.8)	0.244	0.334
Multiple traumas	64 (60.4)	36 (67.9)	28 (52.8)	2.524	0.112
Medical treatment in first 24 h					
Endotracheal intubation	95 (89.6)	48 (90.6)	47 (88.7)	0.101	0.750
Infusion of blood products	92 (86.8)	49 (92.5)	43 (81.1)	2.963	0.085
Hypertonic dehydrating agent	94 (88.7)	47 (88.7)	47 (88.7)	0.000	1.000

GCS = Glasgow Coma Score; SAH = subarachnoid hemorrhage; SDH = subdural hematoma; EDH = epidural hematoma; DAI = diffuse axonal injury.

**Table 2:** PCPC at discharge and at 3-month follow-up, in-hospital mortality and length of stay for the two groups.

	All Patients	DC Group	Non-DC Group	X^2^/z	*p*-Value
PCPC at discharge	(N = 106)	(n_1_ = 53)	(n_2_ = 53)		
1	20 (18.9)	13 (24.5)	7 (13.2)	−2.182	0.029
2	27 (25.5)	12 (22.6)	15 (28.3)		
3	26 (24.5)	14 (26.4)	12 (22.6)		
4	6 (5.7)	3 (5.7)	3 (5.7)		
5	4 (3.8)	2 (3.8)	2 (3.8)		
6	23 (21.7)	9 (17.0)	14 (26.4)		
PCPC at 3 months	(N = 106)	(n_1_ = 53)	(n_2_ = 53)		
1	27 (25.5)	16 (30.2)	11 (20.8)	−1.905	0.057
2	31 (29.2)	15 (28.3)	16 (30.2)		
3	20 (18.9)	10 (18.9)	10 (18.9)		
4	3 (2.8)	2 (3.8)	1 (1.9)		
5	2 (1.9)	1 (1.9)	1 (1.9)		
6	23 (21.7)	9 (17.0)	14 (26.4)		
	(N * = 83)	(n_1_ * = 44)	(n_2_ * = 39)		
Mechanical ventilation (day)	4.0 (1.0, 17.0)	4.0 (1.0, 7.0)	2.0 (1.0, 7.0)	−1.643	0.100
ICU stay (day)	7.0 (4.0, 12.0)	7.0 (3.0, 11.0)	8.0 (4.0, 13.0)	−0.938	0.348
Hospitalization (day)	29.0 (17.0, 46.0)	32.0 (17.5, 49.0)	28.0 (17.0, 46.0)	−0.415	0.678

The PCPC scores were compared using the paired rank sum test. Durations of mechanical ventilation, ICU stay, and overall hospitalization were evaluated with the rank sum test, with an asterisk (*) indicating exclusion of deceased patients from the analysis. Data are presented as frequencies (%) or medians with interquartile ranges (IQR). DC = Decompressive Craniectomy, PCPC = Pediatric Cerebral Performance Category, ICU = Intensive Care Unit.

**Table 3:** Laterality and Localization of Bone Flap Removal in the DC Group.

	DC Group (n = 53)	Mortality	X^2^	*p*-Value	Poor Outcome	X^2^	*p*-Value
Side of the bone flap							
Unilateral	41 (77.4)	6 (14.6)	0.163	0.686 *	21 (51.2)	0.189	0.664 *
Bilateral	12 (22.6)	3 (25.0)			7 (58.3)		
Site of the bone flap							
Supratentorial	45 (84.9)	7 (15.2)	/	0.339 ^#^	24 (52.2)	1.303	0.521 *
Subtentorial	5 (9.4)	1 (25.0)			3 (75.0)		
Both Supratentorial and Subtentorial	3 (5.7)	1 (33.3)			1 (33.3)		

Chi-square test was used in assessing the association between the laterality and localization of the bone flap and outcomes, including mortality and adverse prognosis, in the DC group. *p*-Values marked with an asterisk (*) underwent continuity correction, whereas those marked with a hash (^#^) were analyzed using Fisher’s exact test. Values are shown as frequencies (%). DC = Decompressive Craniectomy.

**Table 4:** Postoperative complications across both groups.

	All Patients (N ^#^ = 65)	DC Group (n_1_ ^#^ 53)	Non-DC Group (n_2_ ^#^ = 13)	X^2^	*p*-Value
Complication					
New hematoma	16 (24.6)	12 (22.6)	4 (30.8)	0.006	0.941 *
Intracranial infection	1 (1.9)	1 (2.6)	0 (0)	/	1.000 ^#^
Cerebral edema	7 (13.2)	5 (9.4)	2 (15.4)	0.001	0.971 *
hydrocephalus	10 (18.9)	9 (17.0)	1 (7.7)	0.844	0.619 *
Herniation	7 (13.2)	6 (11.3)	1 (7.7)	0.000	1.000 *

The chi-square test was employed to evaluate the incidence rates of postoperative complications between the two groups. *p*-Values marked with an asterisk (*) underwent continuity correction, whereas those marked with a hash (^#^) were analyzed using Fisher’s exact test. DC = Decompressive Craniectomy. Values are shown as frequencies (%).

## Data Availability

The dataset used and analyzed during the current study is publicly available at Mendeley Data: Dang, Hongxing (2024), “DC in TBI case-control”, Mendeley Data, V2, https://doi.org/10.17632/vhjnxz7jb4.2 [13].
